# Timing of CD8 T cell effector responses in viral infections

**DOI:** 10.1098/rsos.150661

**Published:** 2016-02-17

**Authors:** Shaun R. Stipp, Abdon Iniguez, Frederic Wan, Dominik Wodarz

**Affiliations:** 1 Institute for Mathematical Behavioral Sciences, University of California, Irvine, CA, USA; 2 Mathematical and Computational Systems Biology, University of California, Irvine, CA, USA; 3 Department of Mathematics, University of California, Irvine, CA, USA; 4 Department of Ecology and Evolutionary Biology, University of California, Irvine, CA, USA

**Keywords:** virus dynamics, cytotoxic T lymphocyte dynamics, CD8 T cells, mathematical models, delay

## Abstract

CD8 T cell or cytotoxic T lymphocyte (CTL) responses are an important branch of the immune system in the fight against viral infections. The dynamics of anti-viral CTL responses have been characterized in some detail, both experimentally and with mathematical models. An interesting experimental observation concerns the timing of CTL responses. A recent study reported that in pneumonia virus of mice the effector CTL tended to arrive in the lung only after maximal virus loads had been achieved, an observation that seems at first counterintuitive because prevention of pathology would require earlier CTL-mediated activity. A delay in CTL-mediated effector activity has also been quoted as a possible explanation for the difficulties associated with CTL-based vaccines. This paper uses mathematical models to show that in specific parameter regimes, delayed CTL effector activity can be advantageous for the host in the sense that it can increase the chances of virus clearance. The increased ability of the CTL to clear the infection, however, is predicted to come at the cost of acute pathology, giving rise to a trade-off, which is discussed in the light of evolutionary processes. This work provides a theoretical basis for understanding the described experimental observations.

## 1. Introduction

CD8^+^ T cell or cytotoxic T lymphocyte (CTL) responses play an important role for the control and clearance of many virus infections. Examples of human pathogens where the role of CTL have been studied in detail include human immunodeficiency virus (HIV) [1–4], as well as hepatitis B [5–7] and C viruses [8–10]. Infections of mice represent a valuable tool for investigating the dynamics of CTL responses against viral infections, and lymphcytic choriomeningitis virus (LCMV) has played an especially important role in this respect [11–16]. CTL can act by inducing lysis of infected cells, and they can secrete soluble mediators that shut down virus replication within cells with a variety of mechanisms [17,18]. Besides controlling infections and thus being beneficial to the organism, anti-viral CTL responses can also negatively impact the host through a phenomenon called CTL-induced pathology [19]. This occurs if the anti-viral CTL response damages the tissue sufficiently to cause disease. The correlates of CTL-mediated control and CTL-induced pathology have been studied in much detail (e.g. [20]).

The rate at which CTL react and respond to antigen is thought to be important for the ability of the CTL to limit virus replication and pathology. This is especially important for CTL-based vaccines. Yet, there is evidence that CTL responses can reach sufficient levels only after a certain time delay, which might compromise the ability of memory CTL to protect the host. In a set of experiments, the presence of LCMV-specific memory CTL failed to protect the animals against peripheral re-challenge, while protection was successful for intravenous infection [21]. It was hypothesized that the inability to protect against peripheral re-challenge was the result of the time it takes for the memory CTL to extravasate into the peripheral tissue. A delay in the rise of a CTL response was also found in a kinetic analysis of data from an HIV vaccine trial [22], and the authors suggested that the delay was the result of an inability of CTL to react against antigen until antigen levels have crossed a certain threshold. CTL delays have also been observed in the context of primary, acute responses. For example, it has been reported that hepatitis C virus outpaces the CTL response by several weeks in chimpanzees [23], and it was hypothesized that this might contribute to virus persistence. The most detailed analysis of the kinetics of acute CTL in both space and time, however, was provided by Frey *et al.* [24], using pneumonia virus of mice (PVM) as a model system. This work demonstrated strong T-cell infiltration into the lung only after maximal virus loads were already achieved, and this also coincided with the development of symptomatic disease in the mice. It was hypothesized that the relatively late infiltration might be important to ensure efficient CTL-mediated clearance of residual disease.

Given that the development of maximal CTL-mediated activity can take time during which the virus infection can spread in the target tissue, strategies aimed at accelerating the development of CTL effector activity are discussed in the literature, especially in the context of vaccination approaches [25]. Here, we explore this topic from a different angle and ask whether it can be adaptive for the host to delay the occurrence of CTL effector activity following the onset of an infection. This is explored with the help of different mathematical models that are designed to describe the dynamics between CTL responses and a viral infection under various assumptions. We find that delayed appearance of CTL effector activity at the site of infection can increase the chances to clear the infection under certain conditions. Although this might increase the extent of pathology that occurs, the ability to clear an infection might be the stronger selection pressure, and an inherent delay in the appearance of CTL-mediated anti-viral activity might thus be adaptive. This analysis is used to interpret the experimental data summarized above, especially the data by Frey *et al.* [24], which motivated our work.

## 2. Results

### 2.1 The simplest model

In the simplest scenario, we do not explicitly model the dynamics of CTL expansion. Instead, we assume that a certain number of effector CTL are ‘placed’ at the site of infection at different time points. They can kill infected cells and die with a certain rate. The dynamics of virus replication are described by standard ordinary differential equations that have been used extensively in the literature [26–29]. Denoting the number of susceptible, uninfected cells by *S*, the number of infected cells by *I*, and the CTL effector population by *Z*, the equations are given as follows:

2.1 $$\begin{array}{rl} \frac{\text{d}S}{\text{d}t} & =\lambda -\text{dS}-\beta \text{SI},\\ \frac{\text{d}I}{\text{d}t} & =\beta \text{SI}-\text{aI}-\text{kIZ}\\ \;\mathrm{and}\;\frac{\text{d}Z}{\text{d}t} & =-\text{bZ}. \end{array}\}$$

This model describes the time evolution of the infection over time. Uninfected cells are produced with a rate λ, die with a rate *d*, and become infected with a rate *β*. Infected cells die with a rate *a* and are removed by CTL with a rate *k*. The free virus population is assumed to be in a quasi-steady state, which is justified if the turnover of the free virus population is much faster than that of the infected cells. Hence, virus load is given by the infected cell population size in this model. Initially, it is assumed that the number of CTL *Z*(0)=0, and that a certain number of CTL are introduced into the system at a defined time threshold, *Z*(*t*=*T*_thr_). Following introduction, the CTL kill infected cells with a rate *k* and die with a rate *b*. The properties of the basic virus dynamics equations are well defined in the literature [28]. An important quantity is the basic reproductive ratio of the virus (*R*_0_=λ*β*/*da*), the value of which needs to be greater than one in order for the virus to establish a successful infection.

**Figure 1.**
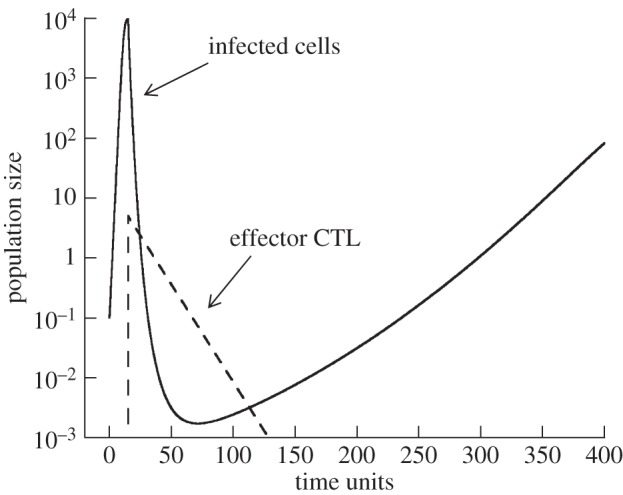
Simulation of model (2.1). The virus population is depicted by the solid line, the effector CTL by the dashed line. Virus growth starts at time zero, and the CTL population is introduced at a later time point. Upon introduction of the CTL, the virus population declines. Owing to the simplicity of the model, the CTL population also declines and is not maintained in the long term. Following the CTL decline, the virus population re-grows. The minimum virus load achieved by CTL-mediated activity is a measure of the effectiveness with which the acute CTL reduce virus load. We examine how this minimum depends on the timing of CTL introduction under different parameter regimes. Parameters were chosen as follows: λ=1, *d*=0.0001, *β*=0.0001, *a*=0.0002, *b*=0.075, *k*=0.25. Initial conditions were as follows: *S*(0)=λ/*d*, *I*(0)=0.1.

Following CTL introduction, virus load is reduced to a certain extent if the CTL-mediated killing rate is sufficiently high. Because the model simply assumes that CTL kill and die after introduction to the site of infection (i.e. they are not maintained in the long term), the virus population will eventually grow back. This means that we investigate acute infection dynamics, and examine the conditions that favour virus clearance during this phase. Chronic infection dynamics, which would require long-term maintenance and stimulation of CTL, are not considered here. The dynamics are demonstrated in figure 1 and crudely represent acute infection dynamics. Because the model is based on ordinary differential equations, the CTL cannot drive the virus population extinct, they can only reduce it to very low levels. The lower the minimum virus load that is achieved after CTL introduction, the higher the chances that the infection will be cleared (owing to stochastic effects in reality). Note that we model the concentrations of cells, and hence, a population size of one does not mark an extinction threshold. The lower the levels to which the infected cells are reduced, however, the higher the chances of virus extinction. An analysis of the exact levels of infected cells at which stochastic extinction becomes likely would require a different modelling approach (stochastic model) and would require a separate study. Therefore, a comparison of the minimum virus load to virus levels that are required to avoid stochastic extinction is not possible in the current framework. This gives rise to one uncertainty that has to be kept in mind when interpreting the results: when comparing the dynamics in different parameter regimes, and observing a reduction of minimum virus load in one simulation compared to the other, this difference is only meaningful if at least one of the minima lies above the extinction threshold.

**Figure 2.**
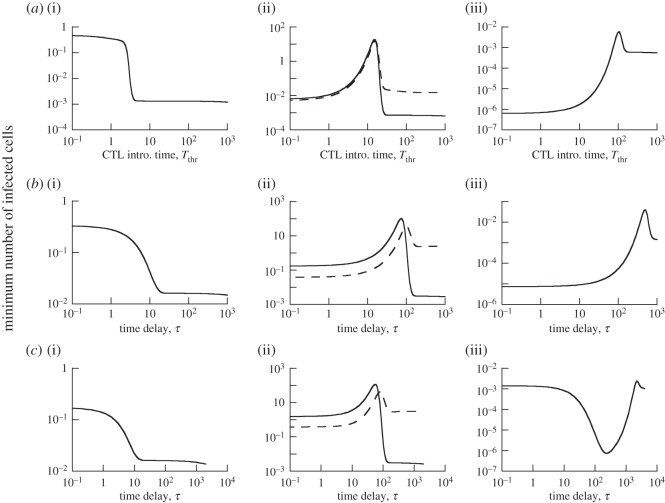
Dependence of minimum virus load on the delay with which effector CTL arrive at the site of the infection, for models ((2.1)–(2.3)). For each delay (or CTL introduction time), the models were simulated, giving rise to a time series similar to the one seen in figure 1. The minimum virus load was determined and plotted. (*a*) Model (2.1), (*b*) model (2.2) and (*c*) model (2.3). For (*a*–*c*), panels (i)–(iii) show the dependence for different parameter combinations. A dashed curve in some of the graphs shows the dependence for an increased turnover rate of the target cell population, with other parameters remaining identical. Specifically, the parameters were chosen as follows. (*a*) λ=1, *d*=0.0001, *a*=0.0002, *b*=0.075, *k*=0.25. Upon introduction, *Z*=5. (*a*(i)) *β*=0.0004; (*a*(ii)) *β*=0.00006, dashed line: λ=100, *d*=0.01; (*a*(iii)) *β*=0.00001. (*b*) λ=1, *d*=0.0001, *a*=0.0002, *b*=0.075, *k*=1, *α*=0.5, *g*=0.01, *r*=1. (*b*(i)) *β*=0.00005; (*b*(ii)) *β*=0.00001, dashed line: λ=100, *d*=0.01; (*b*(iii)) *β*=0.000002. (*c*) Basic parameter same as for (*b*), with the additional parameters *p*=1, *u*=1, *u*_0_=1, *η*=0.5. (*c*(i)) *β*=0.00005; (*c*(ii)) *β*=0.00001, dashed line: λ=100, *d*=0.01; (*c*(iii)) *β*=0.0000005. The initial conditions were as follows: *S*(0)=λ/*d*, *I*(0)=0.1, *R*(0)=0.2, *V* (0)=*V* 0(0)=0, all other variables were set to zero.

Figure 2*a* shows that this CTL-induced minimum virus load is influenced by the timing of CTL introduction, and this is investigated with extensive numerical simulations. In the simulations, we do not assume particular parameter values, but seek to understand the different behaviours that are possible in this model, and how this qualitatively depends on parameters. The CTL introduction times were varied and the minimum virus load was determined. This was done for different parameter combinations, which are defined in the appropriate figure legends throughout the text. Computer simulations indicated that the replication rate of the virus, *β*, had the strongest effect on the relationship between minimum virus load and CTL introduction time. Therefore, the numerical simulations determined this relationship as the parameter *β* was varied from low to high. Parameter values were chosen only for the purpose of illustrating model properties. The emphasis is on determining the different ways in which a delay in CTL arrival can influence the minimum virus load, and not on modelling a specific virus infection.

**Figure 3.**
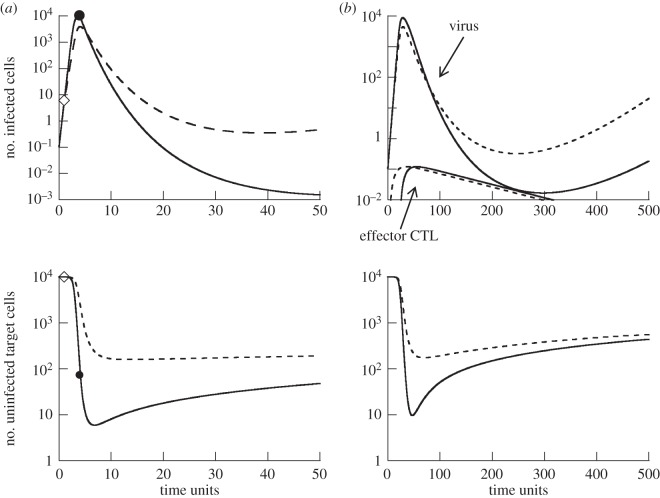
Time-series simulations comparing the dynamics for shorter (dashed line) and longer (solid line) CTL delays. (*a*) Simulation of model (2.1). The diamond symbol indicates the point at which the CTL population is introduced for the short delay scenario, and the circle indicates the CTL introduction time for the longer delay scenario. (*b*) Simulation of model (2.2). The upper panel that shows the virus load curves also shows the dynamics of the CTL effector populations, as this model explicitly describes clonal expansion. Parameters were chosen as follows. (*a*) λ=1, *d*=0.0001, *β*=0.0004, *a*=0.0002, *b*=0.075, *k*=0.25. Initial conditions: *S*(0)=λ/*d*, *I*(0)=0.1. (*b*) λ=1, *d*=0.0001, *β*=0.00005, *a*=0.0002, *b*=0.075, *k*=1, *α*=0.5, *g*=0.01, *η*=0.5, *r*=1. Initial conditions: *S*(0)=λ/*d*, *I*(0)=0.1, *R*(0)=0.2, *V* (0)=*V*_0_(0)=0, all other variables were set to zero.

For relatively high rates of viral replication, we find that minimum virus load becomes monotonically lower for longer CTL delays (figure 2*a*(i)). The reason is that later CTL arrival at the site of infection allows the virus to replicate to higher levels and to reduce the target cell population. Reduced target cell availability lowers the overall rate of virus spread in the presence of the CTL, and thus increases the ability of the CTL to counter the replicating virus. This is shown by computer simulations in figure 3*a*. In the simulation when the CTL are introduced early, they actually fail to stop virus spread, they merely slow it down. Only once the virus population has grown to sufficiently high levels and has sufficiently depleted the target cell population are the CTL able to reduce virus load, leading to a decline of the virus population. In the simulation where CTL are introduced later when virus load is already higher, a decline of the virus population is immediately observed and the rate of decline is faster compared with the early CTL scenario. This is because the target cell population is already reduced to lower levels upon CTL introduction (figure 3*a*). Note that the lower minimum virus load occurs despite the fact that the number of infected cells is higher upon later CTL introduction. This is because the reduced target cell availability significantly increases the decline rate of the infected cell population in this regime.

For lower viral replication rates, a longer CTL delay first leads to a higher minimum virus load (figure 2*a*(ii), solid line). Once the length of the delay crosses a threshold, however, the trend reverses, and a further delay in CTL arrival leads to a lower minimum virus load (figure 2*a*(ii), solid line). This trend is explained by a trade-off that is associated with delayed CTL introduction. On the one hand, a delayed CTL response allows the virus population to reach higher peak levels. If the number of CTL arriving at the site of infection is identical, the minimum virus load tends to be higher if peak virus load is higher. On the other hand, a delayed CTL response and the consequently higher virus load also leads to more target cell depletion. A reduced availability of target cells in turn leads to a faster decline of the virus population in the presence of CTL, as explained above. The balance of these two forces explains the one-humped relationship observed in figure 2*a*(ii), solid line. As can be seen in this figure, a long CTL delay leads to a minimum virus load that is lower than in the absence of a delay (figure 2*a*(ii), solid line). Thus, although an intermediate delay reduces the chance of virus clearance in this regime, a sufficiently long delay results in better clearance chances than no delay. This is not the case if the viral replication rate is even lower (figure 2*a*(iii)). Although a long delay again reduces minimum virus load, the minimum remains higher than in the absence of a delay (figure 2*a*(iii)). Hence, in this parameter regime, the absence of a CTL delay maximizes the chances of virus clearance.

Besides the rate of viral replication, another parameter that can influence the observed pattern is the rate of target cell production. A higher rate of target cell production, λ, makes it more difficult for the virus to deplete the target cell population during growth, and thus reduces the ability of a CTL delay to enhance the chances of virus clearance. This is shown in figure 2*a*(ii), where the dashed line represents the outcome for a higher tissue turnover rate compared with the solid line (i.e. a higher value of λ and *d*, such that the total tissue size remains constant).

To summarize, depending on how efficient the virus is at depleting the target cell population during acute growth, a delay in CTL arrival at the site of the infection may or may not enhance virus clearance. If significant target cell depletion is achieved during virus growth, a CTL delay promotes virus load reduction and thus clearance. Note that in this case, the enhanced ability of the CTL to clear the infection comes at the cost of increased pathology, induced by both the higher virus load, and the target cell depletion that occurs as a result of this delay. Both virus load and target cell depletion have been associated with disease in viral infections [30,31]. These notions will be discussed further below.

### 2.2 Model with clonal cytotoxic T lymphocyte expansion and migration

Here, we introduce a little more realism by explicitly modelling the process of clonal CTL expansion, as well as migration from the lymphoid tissue compartment to the site of virus replication. This is a challenging task. Despite a wealth of information, the processes by which CTL responses are initiated are incompletely understood [32,33], and it is unclear how exactly this process should be described mathematically. In this section, the simplest process will be assumed that is most similar to the scenario explored in the previous section. That is, we assume that in the presence of any amount of antigen in the lymphoid tissue, naive CTL start to undergo rounds of programmed proliferation [34–36], which do not depend on further antigenic stimulation. This proliferation process is assumed to eventually result in the generation of effector CTL that migrate to the site of virus replication. Note that in this model, the rate of CTL activation is not proportional to the amount of antigen present in the lymphoid tissue. Activation is assumed to happen with a constant rate regardless of antigen concentration (as long as antigen is present). This is an unrealistic assumption, but represents the next layer of complexity compared to the previous section. In the next section, we will explore a model where the rate of CTL activation is proportional to antigen and compare the properties.

The model is based on previous work [37] and takes into account the following variables. As before, we denote uninfected and infected cells by *S* and *I*, respectively. Naive, resting antigen-specific CTL are denoted by *R*. Activated CTL that have undergone *i* divisions are denoted by *A*_*i*_. Effector CTL are denoted by *E*. The model is thus given by the following set of ordinary differential equations:

2.2 $$\begin{array}{rl} \frac{\text{d}S}{\text{d}t} & =\lambda -\text{dS}-\beta \text{SI},\\ \frac{\text{d}I}{\text{d}t} & =\beta \text{SI}-\text{aI}-\text{kIE},\\ \frac{\text{d}R}{\text{d}t} & =-\alpha R,\\ \frac{\text{d}A_{0}}{\text{d}t} & =\alpha R-{\text{rA}}_{0},\\ \frac{\text{d}A_{i}}{\text{d}t} & =2{\text{rA}}_{i-1}-{\text{rA}}_{i},\\ \frac{\text{d}A_{n}}{\text{d}t} & =2{\text{rA}}_{n-1}-{\text{gA}}_{n}\\ \;\mathrm{and}\;\frac{\text{d}E}{\text{d}t} & ={\text{gA}}_{n}(t-\tau )-\text{bE}. \end{array}\}$$

The basic virus dynamics equations are the same as in the last section. Resting CTL become activated with a rate *α*. Activated cells divide *n* times with a rate *r* (the factor 2 stems from the division of cells). After the final division, the cells differentiate into effector cells and migrate to the site of infection with a rate *g*. It is assumed, however, that the effector CTL only arrive at the site of infection after a time delay *τ*. Hence, this is a delay differential equation, which was simulated by standard methods, using Matlab. We will explore the effect of varying this time delay on the dynamics of the infection, using the same kind of numerical simulations as described above.

Similar to model (2.1) in the last section, these equations also only describe the acute phase of the dynamics. Long-term maintenance of CTL by continued antigenic stimulation is not included in the model. We examine how the time delay *τ* influences the ability of this acute CTL response to clear the infection. Virus load is reduced by the rising CTL response, but eventually resurges because the CTL response is not maintained. The value of the minimum virus load again correlates with the chances that the infection is cleared.

The results are shown in figure 2*b*, demonstrating that properties are very similar to model (2.1) explored in the previous section. That is, the time delay *τ* plays an important role in determining the minimum virus load. Especially for faster replicating viruses, a delayed appearance of effector CTL at the site of infection can be beneficial, i.e. can lead to a lower minimum virus load (figure 2*b*). For slowly replicating viruses, such a delay tends to be more detrimental (figure 2*b*). The reasons are identical as those discussed in model (2.1). Figure 3*b* shows that the stronger reduction of the virus population with a longer CTL delay coincides with higher acute virus loads and more pronounced target cell depletion, as in model (2.1). As before, this means that successful CTL-mediated clearance can come at the expense of increased pathology.

### 2.3 Antigen-driven clonal expansion of cytotoxic T lymphocyte

Model (2.2) in the last section assumed that CTL undergo clonal expansion at a defined rate in the presence of antigen, but that the rate of clonal expansion is not influenced by the amount of antigen present in the system. Here, we modify this model to include the assumption that the number of resting CTL that are recruited into proliferation is proportional to the amount of antigen present in the lymphoid tissue [34,36,38]. In accordance with data, it is assumed that once CTL have been recruited into the process of clonal expansion, the rate of proliferation or the number divisions the cells undergo are determined by a programme that is not dependent on the amount of antigen. Compared to model (2.2), two additional populations are included. These are the amount of antigen present at the site of infection, *V*, and the amount of antigen present in the lymphoid tissue, *V*_0_. Note that these variables do not represent replicating virus, but viral antigen that is displayed on antigen presenting cells (APCs). The model is thus given by the following set of equations:

2.3 $$\begin{array}{rl} \frac{\text{d}S}{\text{d}t} & =\lambda -\text{dS}-\beta \text{SI},\\ \frac{\text{d}I}{\text{d}t} & =\beta \text{SI}-\text{aI}-\text{kIE},\\ \frac{\text{d}V}{\text{d}t} & =\text{pI}-\text{uV}-\eta V,\\ \frac{\text{d}V_{0}}{\text{d}t} & =\eta V-u_{0}V_{0},\\ \frac{\text{d}R}{\text{d}t} & =-\alpha {\text{RV}}_{0},\\ \frac{\text{d}A_{0}}{\text{d}t} & =\alpha {\text{RV}}_{0}-{\text{rA}}_{0},\\ \frac{\text{d}A_{i}}{\text{d}t} & =2{\text{rA}}_{i-1}-{\text{rA}}_{i},\\ \frac{\text{d}A_{n}}{\text{d}t} & =2{\text{rA}}_{n-1}-{\text{gA}}_{n}\\ \;\mathrm{and}\;\frac{\text{d}E}{\text{d}t} & ={\text{gA}}_{n}(t-\tau )-\text{bE}. \end{array}\}$$

Viral antigen at the site of infection is produced by infected cells with a rate *p*, decays with a rate *u*, and is transported to the lymphoid tissue with a rate *η*. Viral antigen in the lymphoid tissue decays with a rate *u*_0_. Decay of the viral antigen most probably correlates with the disappearance of antigen from the surface of APCs. The CTL activation, proliferation and differentiation process is described in a similar way as in model (2.2). The difference is that now the rate of CTL activation is proportional to the amount of viral antigen in the lymphoid compartment. As before, the appearance of effector CTL at the site of infection, *E*, occurs with a time delay *τ*.

Note that although the rate of CTL activation is proportional to antigen, we still only consider the first round of programmed CTL proliferation and investigate the amount of virus reduction following this phase of the CTL dynamics. This probably corresponds to the initial acute response. The model does not allow for continued stimulation of CTL, which would apply to the long-term dynamics of persistent infections that are not considered here. Thus, following the phase of programmed CTL expansion, CTL simply die as in the models considered above. The same type of analysis as in the last section will be performed.

In models (2.1) and (2.2), there were two main factors that influenced the extent to which CTL reduced the amount of virus during the acute dynamics. These were: (i) the number of infected cells present when the CTL arrived at the site of infection; the larger the number of infected cells, the higher the minimum virus load achieved, and (ii) the number of susceptible target cells that were present when the CTL reduced virus load; a lower number of susceptible cells slows down virus spread in the face of an attack by CTL. In the present model, an additional factor influences the extent to which CTL can reduce virus load during acute dynamics. This is the degree to which CTL become stimulated by antigen. A higher virus load can lead to more CTL being activated and recruited for proliferation. If virus load is kept lower by early CTL activity, however, fewer CTL are recruited to proliferate and to fight the virus. This can make virus clearance more difficult. In the current model, the influence of the CTL delay on infection dynamics represents a balance of these three forces.

**Figure 4.**
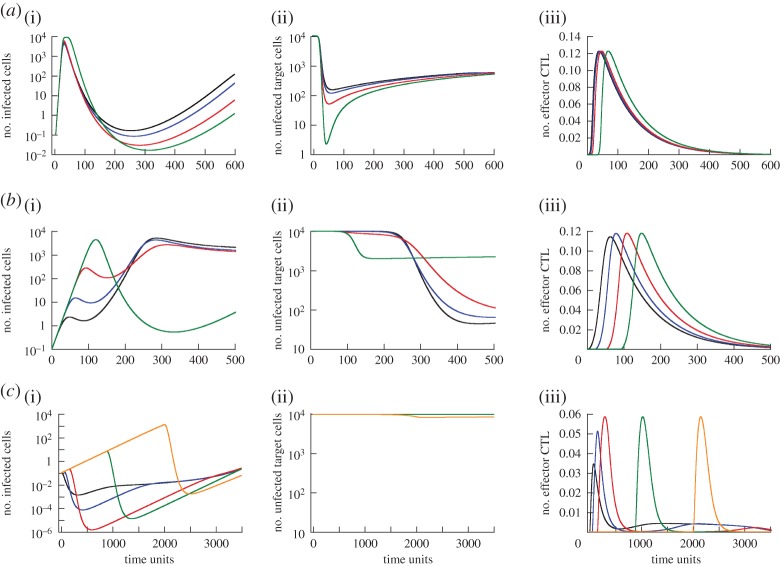
Time series showing the dynamics of (i) infected cells, (ii) uninfected target cells and (iii) effector CTL over time for model (2.3). Panels (*a*–*c*) correspond to the parameter regimes in figure 2*c*(i)–(iii). The different coloured curves in each graph represent different CTL delay times. Colours going from shortest to longest delay time are as follows: black, blue, red, green, orange. These graphs illustrate the reason for the dependencies seen in figure 2*c*. See text for details. For parameter values and initial conditions, see figure 2.

As a result of this, different dependencies of minimum virus load on the delay of effector CTL arrival at the site of infection are observed. This is shown in figure 2*c*, and discussed as follows. (i) In the first pattern, delaying the arrival of effector CTL reduces minimum virus load monotonically, similar to the observations in models (2.1) and (2.2) (figure 2*c*(i)). The reason can be twofold in the current model: as before, a longer delay leads to more pronounced target cell depletion (figure 4*a*(ii)). In addition, however, the longer delay and higher virus loads can recruit more CTL into proliferation, which also reduces minimum virus load. (This is not the case in the simulation in figure 4*a*, but can be observed.) Either one of these mechanisms alone or a combination of both can explain the observed trend. (ii) This pattern is also similar as before, and explained by the same mechanism. First a longer CTL delay leads to a higher minimum virus load, followed by a reduction of minimum virus load to relatively low levels (figure 2*c*(ii)). The reason is again the trade-off between increased virus load and reduced target cell availability for longer delays (figure 4*b*(i)(ii)). In this regime, the size of the CTL effector population is not significantly affected by the delay and hence does not modulate outcome (figure 4*b*(iii)). As in previous models, the tissue turnover can influence whether a longer delay leads to a lower minimum virus load or not compared to the absence of a delay (figure 2*c*(ii), solid versus dashed line). (iii) According to this pattern, a delay in CTL arrival first leads to a decline of minimum virus load, but subsequently, a further delay increases minimum virus load. The reduction in minimum virus load is brought about by an increase in the number of CTL effectors that are generated in response to increased antigenic stimulation for longer delays (figure 4*c*(iii)). The reason this occurs in this parameter regime is that the viral replication rate is relatively slow. Hence, longer delays allow the virus more time to achieve levels that result in the activation and proliferation of more CTL. The subsequent rise of minimum virus load occurs because a further delay does not lead to a further increase in the number of effector CTL (figure 4*c*(iii)). This is because all CTL available for activation had already been activated. At the same time, however, the further delay leads to higher peak levels of infected cells (figure 4*c*(i)), explaining why the minimum virus load becomes higher. Although the number of susceptible target cells is reduced with these long delays (figure 4*c*(ii)), this is outweighed by the higher peak virus loads which occur during the prolonged absence of CTL-mediated activity. This demonstrates how the intricate balance of the various driving forces can determine the exact effect of the CTL delay.

Which pattern is observed is determined by parameter combinations in a complex way. In general, however, simulations indicate that a faster rate of viral replication tends to create conditions under which a delay in the CTL effector response promotes reduction of virus load and thus viral clearance. The rate at which the tissue turns over can also have an important influence, as before. In addition, the number of pre-existing resting CTL precursor cells can play a role. As pointed out above, a longer CTL delay and the consequently higher virus load can activate more precursor CTL, leading to higher effector cell populations. If the precursor CTL population is large, this effect is stronger compared with a scenario where fewer CTL precursors exist.

## 3. Discussion and conclusion

The analysis presented here has shown that the relationship between the timing of CTL responses and their ability to reduce virus load during acute infection is complex and not straightforward. Over wide parameter regions, a delay in the appearance of effector CTL at the site of infection can lead to a stronger reduction of virus load and thus to a higher chance of the acute CTL response to clear the infection. This occurs because a delayed CTL effector response leads to higher acute virus loads, and this can (i) recruit more resting CTL into proliferation and (ii) reduce the number of susceptible target cells. Both effects promote CTL-mediated reduction of virus load. This enhanced ability to reduce virus load, however, is suggested to come at the cost of higher pathology during acute infection, as a result of the higher amount of virus that is present.

These insights improve our previous understanding in a variety of ways. First, the model has shown that a fast response might not always be an optimal response. Second, the model has shown that either a fast or a delayed response might be advantageous under different parameter combinations, which have been defined. Third, the model indicates that if a delayed CTL response promotes virus clearance, this might come at the cost of increased acute pathology.

A difficulty in our investigation was that the dynamics could only be explored with numerical simulations. Therefore, we varied the value of each parameter over extensive ranges and recorded the observed patterns. In these explorations, we only saw the patterns reported here. The differences in parameter values that gave rise to the different types of dynamics are defined in the appropriate figure legends in order to get an idea about the order of magnitude changes that are required to see a switch from one pattern to the other. We currently do not have information about the biologically realistic parameter ranges for the infections discussed here, so we aimed to catalogue the model behaviour, in general, in order to understand what types of dynamics this model can give rise to. As discussed further below, parameter measurement in the context of specific infections will be the next important step in order to determine whether the possible explanations discussed here indeed apply.

Our model assumed a delay in the arrival of CTL at the site of infection, which was modelled by assuming that it takes a certain amount of time for differentiated effector CTL to migrate to the location of virus replication. A similar effect would be observed if it was assumed that CTL only become activated once virus load has risen above a critical threshold. This would also result in a situation where the effector CTL only start acting once virus levels have grown to higher loads [25]. Indeed, this explanation for a CTL delay was evoked in a kinetic analysis of HIV infection data in the context of a vaccine trial [22]. While the biological mechanism would be different, the net effect is similar.

As mentioned in the Introduction, a study that examined the spatio-temporal dynamics of CTL responses against PVM provided the strongest evidence that CTL responses *in vivo* can arrive at the site of infection with a significant delay [24]. Indeed, this experimental study motivated our work with the aim to offer a possible explanation for these results. The CTL responses were observed to infiltrate the lung only after maximal virus load was achieved, and this coincided with the development of symptomatic pathology in the mice. The authors of this study speculated that these characteristics might enhance the ability of the CTL response to clear the infection. Our model allows us to give these observations a theoretical underpinning. The model suggests that allowing the virus to grow to near maximal levels might indeed enhance the ability of the CTL to clear the infection, and it identified the mechanisms by which this is achieved: target cell depletion minimizes the ability of the virus to spread in the face of CTL-mediated activity, and higher virus loads recruit more naive CTL into proliferation which can make sure that any remaining virus is removed. The model further suggests that there might be a general trade-off between the level of acute virus load/pathology and the ability of a CTL response to clear a virus infection. While keeping acute virus load lower will reduce the degree of pathology, it can reduce the chances to clear the infection in the long term, which could be costly. Note that the pathology which results from CTL delays in our model results from the virus depleting the target cells. If the virus is less cytopathic, the CTL response can similarly cause pathology in the model, as was the case in the study by Frey *et al.* [24]. In the light of our theory, it can thus be speculated that the response against PVM has evolved the observed characteristics in order to maximize the chances that the infection is successfully resolved.

In the context of vaccines, it is thought that delays in CTL dynamics upon challenge can present an obstacle for successful CTL-mediated protection against infection [39,40]. The goal is often to maximize the speed with which anti-viral CTL appear at the site of infection. For infection prevention, this is indeed the best strategy. If the goal is to prevent establishment of infection through boosting of the CTL response, a sufficient number of effector CTL needs to be present at the site of infection at the time of virus entry, such that the basic reproductive ratio of the virus is less than one. In a natural setting, however, the goal of CTL might be less to prevent infection, but to clear a replicating virus from the host [40]. According to our model, this can be promoted by a delayed appearance of effector CTL at the site of infection, and the organism might have evolved such delays in order to be more effective in this respect. Therefore, when trying to accelerate the rise of a CTL response through vaccination and therapeutic approaches, we might be acting against the system’s naturally evolved strategy, which might explain some of the difficulties encountered when aiming to boost CTL to prevent human viral infections.

Obviously, the ability of CTL to clear an infection is influenced by many factors. What is clear from our analysis is that delay is one of those factors. Whether the immune system has specifically evolved to delay a CTL response in order to maximize its potential to resolve infections cannot be determined by our approach. Delayed CTL responses that are observed could be explained by alternative hypotheses. In addition, as our analysis has indicated, while a CTL delay can enhance the ability of CTL to reduce acute virus load over wide parameter regions, in other parameter regimes the absence of a delay results in the strongest reduction of virus load. This depends on the parameters, such as the replication kinetics of the virus, or the turnover rate of the tissue that is infected. This in turn varies from one infection to another. Therefore, it is unclear whether a ‘universal’ delay of CTL against viral infections has evolved for the benefit of the host. It might have evolved if a delay results in the better resolution of infections that exert significant selection pressures on the host, and this could mean that CTL responses against other infections that exert less selection pressure on the host are less effective than they could otherwise be. While these uncertainties remain, our study does highlight that a CTL delay can have a significant influence on the ability of CTL to clear virus infections, and that this could come at the cost of increased transient pathology, which is consistent with data [24]. This will hopefully motivate further research that examines this more closely, both from an evolutionary theory and from a virological point of view. From a theoretical perspective, the modelling can be expanded in order to explicitly account for spatial effects, as these probably play a role in tissues like the lung, which was explored by Frey *et al.* [24]. From an experimental point of view, it will be interesting to perform a comparative study that examines the spatio-temporal dynamics of CTL responses to different viral infections and tissues. Our theory predicts that a CTL delay may or may not be beneficial for the host depending on the exact conditions. It would thus be relevant to investigate whether differences in CTL delays exist among different viral infections. In this context, it will be very important to measure crucial parameters in the viral-host systems considered. Our current analysis investigated the model behaviour in general, exploring how different results are observed in different parameter regimes. Such an understanding is important to interpret available observations, and forms the basis for running the model with specific parameter values, which are currently not available for the experimental systems discussed here. If differences exist in the amount of CTL delay observed among various virus–host systems, the model suggests that these are probably explained by variations in the viral replication kinetics as well as the rate of tissue turnover. In general, however, it would be necessary to measure as many kinetic parameters as possible such that it can be determined whether the explanations derived from the model indeed apply to the experimental systems under consideration.

When investigating the reasons for observed CTL delays further and when using our theoretical insights as guidance, it is important to remember that our mathematical work does not provide a proven explanation for the data that exist so far. Our models are intriguing in that they indicate that CTL delays can have a profound impact on the ability of CTL to clear the infection and that a trade-off might exist between ability to clear and the extent of acute pathology. These are testable predictions, as discussed above. Also, as mentioned earlier, it is important to remember that other factors might also contribute to explaining the existence of CTL delays, and that indeed multiple factors might work together to account for the observed data. We have argued that a delayed CTL response might be adaptive for the host in certain parameter regimes. In this context, it is important to be aware of possible constraints that might limit the speed with which CTL responses can rise. The activation of CTL responses involves a complex interplay among CTL, helper T cells, and components of the innate immune system. These interactions might take some time, leading to an inherent delay that might not be possible to overcome. Whether this is indeed the case remains open to investigation. A previous theoretical study [39] argued that the effector/target ratios only become favourable for immune control around peak virus load, a result that might point in this direction. This model, however, assumed that the rate of CTL expansion in the initial stages of infection is proportional to the amount of antigen, which might not be supported by the data on programmed CTL proliferation; hence, this notion merits further investigation. Finally, it is important to point out that model results depend on the assumptions underlying the models, which are clearly spelt out here. In addition, the same biological processes can potentially be formulated mathematically in different ways, and hence the robustness of results that emerge from models needs to be investigated. We have considered three models with different levels of biological complexity, and they all point towards the same basic results, which indicates that these results are indeed robust.
